# Eating Patterns of Young Women (18–25 y) with Overweight and Obesity: A Preliminary Investigation

**DOI:** 10.3390/nu15071652

**Published:** 2023-03-28

**Authors:** Isabel E. Young, Natalie Crino, Katharine S. Steinbeck, Helen M. Parker

**Affiliations:** 1 Sydney School of Health Sciences, Faculty of Medicine and Health, University of Sydney, Camperdown, NSW 2006, Australia; 2 Charles Perkins Centre, University of Sydney, Camperdown, NSW 2006, Australia; 3 Discipline of Child and Adolescent Health, Department of Adolescent Medicine, The Children’s Hospital at Westmead, Westmead, NSW 2145, Australia

**Keywords:** young women, dietary patterns, distribution of energy intake, obesity, diet quality

## Abstract

Overweight and obesity impact up to 40% of young women in Australia; however, young women are challenging to recruit to research and are rarely the focus of weight loss interventions. This study aimed to examine dietary patterns in young women (18–25 years; BMI > 25 kg/m^2^). An analysis of participants’ (mean age: 22.6 year; BMI: 32.2 kg/m^2^) 3-day food records found young women with overweight/obesity consumed a diet characterised by total energy intake of 9174 (2526) kJ/day, with the first meal at 9:12 am (range: 4:30 am–12:40 pm), the last at 10:43 pm (range: 2:40 pm–2:00 am), and an average eating window of 11.5 h. Young women had poor quality diets, which did not meet dietary recommendations for most core food groups, and high intake of refined carbohydrates. They also reported consuming at least one takeaway meal per day and >30% of total energy intake was from discretionary items. The findings showed that young women with overweight or obesity consume most of their energy intake in the afternoons and late into the evenings and have poor-quality diets with high-discretionary intake, each of which have been shown in previous work to be associated with increased weight and risk of metabolic comorbidities. While these findings require further examination in larger groups with both qualitative and longitudinal data collection to verify the impact of these eating patterns on weight maintenance, the eating behaviours identified here may present a suitable target for novel weight loss interventions in young women, who are an understudied population group in need of tailored weight management solutions.

## 1. Introduction

Overweight, obesity, and weight gain in young adulthood increase the likelihood of overweight/obesity in adulthood and the consequent risk of lifetime chronic disease [1,2,3], and for young women, obesity during pregnancy has been linked to poor health outcomes in offspring [4,5]. Young adults aged 18–25 years are particularly vulnerable to weight gain and are gaining weight faster than their older counterparts [6,7,8]. Young adulthood is characterised by significant life changes, such as leaving the family home, resulting in increased independence, particularly with regards to food choices [1,9]. Young adults living away from the family home, learning to balance the many demands of independent living and often with limited cooking and meal preparation skills, are at risk of poor-diet quality, a contributing factor to risk of weight gain [2,3,9].

Preventing weight gain and treating overweight/obesity in young adulthood, especially in young women, is of great importance for long-term health outcomes for the population at large. However, young women are challenging to recruit to research studies [10]; thus, while we know the potential effects of excess weight in young women, this population remains an under-studied group for weight management [9,11], and few dietary interventions are tailored for their unique life stage even though tailoring is considered an important feature of weight loss programs for young women [12].

Energy-restricted diets are typically the first-line treatment for weight loss and long-term weight management [9,13,14] and, being relatively inexpensive compared to other treatment modalities, are accessible to young people who may not be as financially secure as their older counterparts. However, evidence shows that energy restriction may not be an effective weight management tool for some individuals, and the relationship between diet and weight management is complex [15,16].

Recent research into dietary factors influencing bodyweight have expanded beyond dietary energy restriction into dietary patterns, including diet quality and the distribution of total energy intake across the day [17,18,19]. Evidence shows that daily energy intakes characterised by small morning meals, larger evening meals, and energy-dense snacking into the late evening are associated with the presence of overweight and obesity [20]. In addition, interventional studies have shown that distributing energy intake with a focus on distributing total energy intake towards the beginning of the day (e.g., consuming the bulk of total daily energy at breakfast and lunch) is associated with enhanced weight loss [21,22,23,24]. However, while the evidence supporting the earlier weighting of energy intake is promising, such studies have recruited middle-aged or older adults [17,19], with young adults a relatively under-studied population group.

Dietary patterns, including the distribution of total energy intake across the day, have been linked to weight management and metabolic disease; however, these patterns have thus far been explored mainly in middle-aged and older adults. The aim of this study was to examine the dietary patterns of young women with overweight and obesity, in particular the distribution of total energy intake across the day in conjunction with other factors known to impact health, such as diet quality, physical activity, and alcohol intake. In characterising dietary patterns that may be associated with obesity in young women, this study may inform or lead to the development of tailored dietary education and weight management strategies specifically for young women.

## 2. Materials and Methods

### 2.1. Recruitment

A convenience sample of young women with overweight and obesity were recruited as part of a larger weight management intervention approved by local health district human research ethics committees (HREC/16/RPAH/269—X16-0218). Participants were recruited from metropolitan Sydney via paid social media and commercial back of toilet door advertising, flyers on tertiary education campuses, and advertising through online news bulletins, such as workplace newsletters and intranet sites.

For the current study, data were included and analysed from participants with complete data of interest at baseline, the details of which are given below.

### 2.2. Eligibility

Eligible young women were aged 18–25 years with a BMI > 25 kg/m^2^ and reported being weight stable for a minimum of 3 months. The young women needed to be otherwise healthy with no underlying health conditions; however, exceptions were made for polycystic ovarian syndrome, and if taking medications known to affect weight, the dose prescribed had to have been stable (unchanged for >6 months). Women who were smokers, pregnant, lactating, or <6 months post-partum were excluded.

### 2.3. Procedure

Participants provided demographic information, including age and occupation, and were asked to complete a 3-day food diary and the Global Physical Activity Questionnaire (GPAQ), as described below. Quantities of foods and beverages were either estimated in household measures or weighed using kitchen scales, as per the preference of the participant. Instructions on measuring different foods and beverages were also provided to participants by the study dietitian and were included as part of the food diary. After completing the above, participants met with the study dietitian who ensured that the food diary was completed correctly, including extra details as required during the interview. Participant’s weight and height were measured at this time, in light clothing and without shoes, using an electronic digital platform scale (PW-200KGL, Thebarton, Adelaide, Australia) to the nearest 0.01 kg and a stadiometer (213 portable stadiometer; SECA, Hamburg, Germany) to the nearest 0.1 cm, respectively.

### 2.4. Data Analysis

Patterns of eating were assessed using the 3-day food diary. Food diary data were collated and analysed using FoodWorks (FoodWorks 10 Professional, v10.0. Brisbane, Australia: Xyris Pty Ltd., Brisbane, Australia, 2019), which uses nutrient information from the AUSNUT 2011-13 nutrient database. This provided an assessment of intake at each eating or drinking occasion across the day and daily total intake of macronutrients, micronutrients of interest, energy, and number of serves of core food groups. An eating or drinking occasion was defined as any time that equated to intake of energy (i.e., intake of water alone did not count as an eating or drinking occasion). Serves of food groups and discretionary items were calculated using the energy per serve provided by the Australian Guide to Health Eating (AGHE) [25]. Diet quality was assessed by comparing the macronutrient composition and number of serves of core food groups to the national recommendations in the AGHE [25]. Intake of iron and calcium were also calculated using FoodWorks as nutrients of interest in young women and compared to the recommended daily intake (RDI) and estimated average requirement (EAR), as set by the Australian Nutrient Reference Values (NRVs) [26].

Habitual physical activity was surveyed using the GPAQ [27]; total metabolic equivalent of task minutes (MET-minutes) of physical activity per week and MET-minutes of leisure time physical activity (physical activity conducted recreationally and not part of travel or occupation) per week were calculated. Participants’ total physical activity was then compared to current physical activity guidelines; participants were characterised as to whether or not they achieved at least 500 MET-minutes of physical activity/week, which is equivalent to 2.5 h of moderate intensity physical activity of 1.25 h of vigorous intensity physical activity, which are the current recommendations for maintaining health and disease prevention [28].

### 2.5. Statistical Analysis

Data were analysed using Statistical Package for the Social Sciences (SPSS, IBM Corp. Released 2021. IBM SPSS Statistics for Windows, Version 28.0. Armonk, NY, USA: IBM Corp.). Normally distributed data are presented as mean (standard deviation, SD), and non-normally distributed data are presented as median (range). Spearman’s correlations were conducted to examine relationships between BMI and other variables.

Independent samples *t*-tests were used to compare differences in demographic, anthropometric and dietary variables between alcohol drinkers and non-drinkers, and between participants who did or did not meet total physical activity guidelines. Participants who engaged in leisure time physical activity (activity completed recreationally and excluding activity done as part of travel or occupation) were also compared to those who did not, irrespective of whether physical activity guidelines for total physical activity were being met. These groups were compared for BMI, age, timing of first and last meals, eating window and diet quality, including macronutrient distribution, intake of core food groups, and intake of discretionary foods.

## 3. Results

A total of 28 young women (age: 22.6 (2.1) year) with overweight or obesity (BMI: 32.2 (4.3) kg/m^2^) completed data collection. Two-thirds (67.9%) of participants were currently enrolled in tertiary education; the remainder were employed in full-time work. Participants engaged in an average of 2930 (3778) MET-minutes/week of total physical activity and 360 (range: 0–3120) MET-minutes/week of leisure time physical activity, with 46.4% meeting the recommendations for total physical activity. Interestingly, leisure time physical activity but not total physical activity was found to be negatively correlated with BMI (ρ = −0.435, *p* = 0.021).

### Eating Behaviour and Diet Quality

The analysis of 3-day food diaries (Table 1) showed that, on average, the young women began consuming energy-containing foods/beverages at 9:12 am with their last meal at 10:43 pm. The earliest eating/drinking occasion reported was at 4:30 am and the latest at 2 am. The mean eating window across the day was 11.5 (2.3) h with 78.5% of participants having days where they ate beyond 8 pm and 53.6% beyond 10 pm. On average, participants reported five eating occasions over the day and a total energy intake of 9174 kJ/day.

**Figure 1:**
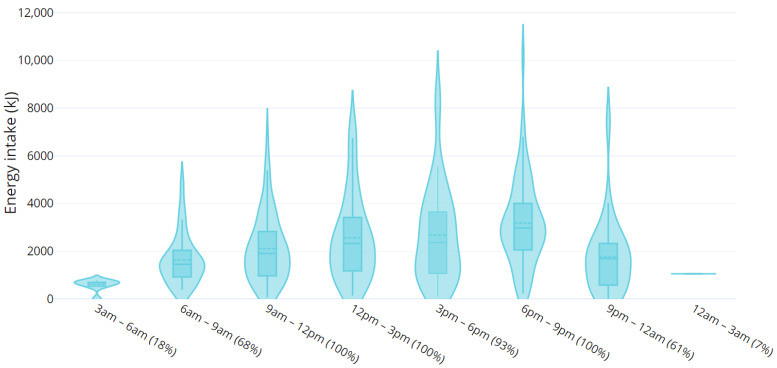
Energy intake across the day. Distribution of energy intakes during 3 h intervals across the day and the percentage of participants who reported at least one eating/drinking occasion within each 3 h interval. The bulbous section(s) in each column represents the area(s) of highest density of reported energy intakes with energy intakes reported by fewer participants represented as thin/less bulbous areas of each column. The percentage represents the proportion of participants reporting consuming energy during that time period.

**Figure 2:**
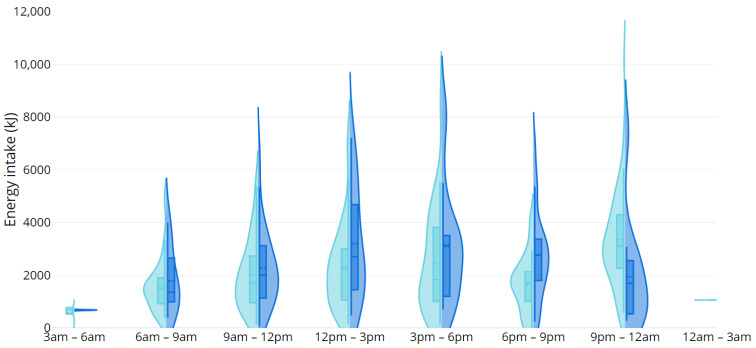
Distribution of reported energy intake during 3 h intervals across the day weekdays (light blue) vs. weekend (dark blue) days.

Distribution of total energy intake across the day can be seen in Figure 1. All participants consumed energy-containing food or drink between 9 am and 3 pm and 6 pm and 9 pm. The time period with the highest energy intake of the day was 6–9 pm with mean (SD) of 3180 (1822) kJ consumed during that time. There were differences in energy intake on weekdays compared to weekends (Figure 2): weekday energy intake was highest between 6 and 9 pm (3358 (1852) kJ), whereas energy intake over the weekend peaked earlier in the day at 12–3 pm (3198 (2113) kJ) and remained high from 3 to 6 pm (3161 (2250) kJ), tapering only slightly at 6–9 pm (2759 (1723) kJ). Young women also reported consuming energy-containing food/beverages later into the night on weekdays compared to weekends, with the latest eating occasion being at 2 am and midnight, respectively (Figure 2).

Overall, diet quality was poor with participants reporting a high intake of fat and saturated fat; mean percentage of total energy intake contributed by these macronutrients being 36% and 17.8%, respectively, well above the World Health Organisation (WHO) guidelines of 30% and 10% [29]. Protein accounted for 19.4% of total energy intake, and carbohydrates accounted for 39.8%. Intake of core food groups was below the minimum recommendations except for grains; however, the majority of grains comprised starchy or refined grains with only 29% of grains consumed being wholegrains. Twenty-five percent of participants consumed less than 1 serve of dairy per day, 64% consumed less than 1 serve of fruit, and a third (32%) of the young women consumed less than half the recommended serves of vegetables. Only 7% of participants (*n* = 2) met the RDI for iron of 18 mg/day, and 82% met the EAR of 6 mg/day with the average intake for the group being 11.54 (5.78) mg/day. Intake of calcium was also low with only 25% of participants (*n* = 7) meeting the RDI of 1000 mg/day, and 46% (*n* = 13) meeting the EAR of 840 mg/day; mean calcium intake was 804.8 (283.9) mg/day. Correlations found that vegetable (*p* = 0.046) and proportion of wholegrain intake (*p* = 0.044) were weakly negatively associated with BMI (ρ = −0.380 and −0.384, respectively); no other variables were found to be significantly correlated with BMI. Discretionary foods/beverages contributed 36.8% of total energy intake with participants reporting a mean of six serves per day (SD: four serves), which is above the current recommendations of zero serves per day. Participants consumed on average one sugar-sweetened beverage (SSB) and one non-nutritive-sweetened beverage (NNSB) daily. Alcohol intake was variable with 32% (*n* = 9) of participants reporting consuming alcohol during their 3-day food diary; the average intake among the young women who reported drinking alcohol was 1.8 standard drinks/day with 0.6 (1.1) standard drinks on average for a single day across the whole group (drinkers and non-drinkers).

When comparing participants who reported consuming alcohol (*n* = 9) to those that did not (*n* = 19), there were no significant differences in total energy intake, intake of core food groups, or timing of intake. However, there was a significant difference in the number of serves of discretionary foods consumed per day with participants who consumed alcohol reporting a higher intake of discretionary serves than non-drinkers (drinkers: eight (3.5) serves; non-drinkers; five (2.8) serves per day (not including alcohol); *p* = 0.042). When comparing participants who met total physical activity guidelines to those that did not, there were no differences seen across timing of intake, BMI, or diet quality. However, when looking at leisure time physical activity alone (physical activity which is not undertaken as part of travel or occupation), it was found that participants engaging in some level of leisure time physical activity had a lower BMI than those who did not (recreational activity: 30.6 kg/m^2^; no recreational activity: 34.5 kg/m^2^; *p* = 0.013). This was found to be irrespective of whether the leisure time physical activity reported met or did not meet the leisure time physical activity guidelines of 500 MET-minutes per week.

The young women reported consuming at least one non-home-prepared main meal per day (range: 0–3), which was either consumed as takeaway or at a restaurant/food venue with 18% of participants consuming on average more than one non-home-prepared meal per day. These meals were most often consumed in the evening at dinner. The young women also reported a high intake of total individual discretionary items, independent of the total number of serves of discretionary foods/beverages. These items were consumed more frequently later in the day with 64% of all discretionary items being consumed between lunch and going to sleep and 24% being consumed between the evening meal and going to sleep. Discretionary items consumed at dinner were the most energy dense (discretionary items consumed at dinner contributed 1812 (1301) kJ, including main meals comprised of discretionary food). The discretionary items which were consumed most frequently (irrespective of serve size) by participants were sweet baked goods (16.9% of total discretionary items), chocolate (15.9%), processed meats (8.5%), and hot chips/deep fried foods (7.5%) with each eating occasion at which discretionary food/beverages were consumed contributing an average of 1226 (1095) kJ to total energy intake.

## 4. Discussion

The aim of this study was to examine dietary patterns of young women with overweight and obesity, in particular the distribution of total energy intake across the day. Young women with overweight and obesity were found to distribute their energy intake more heavily in the late afternoons and evenings as compared to earlier in the day. They reported eating for almost half of the calendar day with most young women eating beyond 9 pm and through to midnight with a greater intake of energy dense discretionary food/drink items and non-home-prepared meals in the evenings compared to earlier in the day. The young women also had poor-diet quality, not meeting the recommendations for any core food groups except for grains, and even then, consuming mostly refined grains rather than wholegrain foods. They also reported high intakes of takeaway and discretionary items and participated in limited recreational physical activity with less than half of the young women meeting leisure time physical activity guidelines.

The distribution of energy intake towards the afternoon and evening has become the norm in many societies, particularly in western cultures where dinner (the evening meal) is seen as the main meal of the day [30]. The evening meal is also commonly seen as a social occasion, holding emotional and social importance, which can lead to greater food and energy consumption [30,31]. The participants in the current study commonly reported consuming the largest portion of their total daily energy intake between 6 and 9 pm, reflecting this same attitude reported in the literature of the evening meal being the largest or most important meal of the day [30,31]. This pattern of energy distribution was particularly evident on weekdays compared to weekends with the young women consuming greater amounts of energy in the evenings on weekdays compared to during the middle of the day and early afternoon on weekends. Reasons for this may include the restrictions placed on individuals by common working hours or ‘business hours’, which leads to increased evening intake as this is when individuals have the most time to prepare and consume food [32]. While it is convenient to capitalise on the greater availability of ‘free’ (non-work) time in the evening to prepare and consume larger and more elaborate meals, this pattern of eating has been linked with metabolic disease and increased risk of obesity in middle-aged and older adults [18,20]. Research has also shown that encouraging a shift in energy intake to favour the morning can result in greater weight loss in older adults engaging in weight loss interventions when compared to equally energy-restricted diets that do not emphasise earlier distributed energy intake [21,23,24]. Similar interventions may have clinical value in the female young adult cohort.

The larger total energy intake in the late afternoon and evenings may also be driven by the higher intake of non-home prepared meals during these times. Dinner was the most common meal, which was not prepared at home, with participants instead having takeaway or consuming their meals at a restaurant. Meals eaten out of home are typically more energy dense with higher saturated fat, refined carbohydrate, and salt content compared to self-prepared meals [33,34,35]. The consumption of non-home-prepared meals has increased significantly in recent years and with regular takeaway and restaurant meals becoming increasingly normalised [36,37]. The increase in meal delivery services has exacerbated this, increasing the convenience with which one can access takeaway meals. Additionally, many meal delivery platforms now operate on mobile devices of which young people are the largest users [38,39]. In addition to convenience, the increased intake of meals not prepared at home may also be driven by time constraints of young women and changes in societal values, which may lead to decreased cooking skills [37,40]. The high intake of non-home-prepared meals by young people highlights a need for further research to better understand the context in which these meals are procured and consumed. A limitation of this study is that the participants’ reportings in the food records did not make it clear whether meals were eaten at a restaurant or as takeaway, and additional information regarding whether these meals were eaten alone or as part of a social occasion was not collected. This information could further inform what education is needed to best improve the diets of young women with overweight and obesity.

In addition to the high intake of non-home-prepared meals, the high-energy intake in the evenings may be related to intake of energy-dense discretionary foods. Over the course of the average day, the young women in this study reported the highest intake of discretionary items in the evenings, both by number of items and by total energy intake from discretionary items. This high intake of discretionary items also contributed to the overall poor-diet quality. Higher intake of discretionary items is associated with lower intakes of core food groups in the diet, resulting in decreased intake of essential nutrients [41,42]. Higher intake of core food groups has also been found to be associated with improved health outcomes, particularly vegetables and wholegrains, which were found to be negatively associated with BMI in the current study and which have been linked to decreased risk of cardiovascular and other metabolic diseases [20,43,44]. Similar to other studies, in addition to high-discretionary intake, participants were also not meeting current recommendations for core food groups or key micronutrients for young women’s health, namely calcium and iron [41,42]. The only exception was for intake of grains; however, the grains consumed by the young women were mostly refined, again decreasing their overall diet quality. Additionally, whilst the majority of participants did not report consuming alcohol during the three days of data collection, those that did reported an average of 1.8 standard drinks per day, which is above current guidelines for alcohol intake for women [25]. The consumption of alcohol was also shown to be associated with a significantly higher intake of discretionary items per day, which was only partly accounted for by the intake of alcohol. This pattern of eating is in line with current data in young adults where they have been found to have poor-diet quality, characterized by low intake of core foods and high-energy density [42]. Furthermore, diet culture and the attraction of young women to “quick-fix” diets to help with weight loss may also be contributing to their low intake of core foods as they can encourage removal or avoidance of whole food groups [45,46]. This can further exacerbate poor eating habits, which, once formed during the early years of adulthood, can continue into middle- and late-adulthood and further increase the risk of chronic disease.

Encouraging leisure time physical activity in the group may also provide additional benefits to metabolic health [47]. Whilst the group reported high levels of total MET-minutes/week, less than half of the young women met the current physical activity guidelines, which outline a goal of at least 500 MET-minutes total physical activity per week. However, when exploring leisure time physical activity alone, it was seen that participants who engaged in some level of leisure time physical activity, irrespective of whether they were meeting current physical activity guidelines in leisure time, had a significantly lower BMI than participants who engaged in no leisure time physical activity at all. Furthermore, while these are cross-sectional data, it is noteworthy that increased leisure time physical activity was also found to be negatively associated with BMI. This supports current evidence, which has shown that whilst high levels of total activity are beneficial, intentional leisure time or recreational physical activity has additional health benefits beyond those gained from the physical activity done as part of work or commuting [47]. Levels of physical activity in females in particular are negatively impacted by the numerous life transitions that occur during adolescents and into young adulthood [48]. Therefore, encouraging and supporting young women to continue recreational and leisure time activity beyond their schooling years and during this crucial time of transition to adulthood may benefit their metabolic health and weight management.

Each of these factors discussed above, the evening meal as the main meal of the day, non-home-prepared meals becoming increasingly normalised, energy-dense discretionary foods, alcohol intake, and low leisure time physical activity, are individual potential upward drivers for bodyweight in young adults. However, the mechanisms linking each component to weight change may have a variety of pathways by which they act. Many of these factors are associated with altered or suboptimal nutrient density of the overall diet, which may affect appetite and subsequent food/beverage intake. This body of work examining macronutrient drivers of appetite is important to consider here, for example the protein-leverage hypothesis, whereby low-protein density of the diet has been shown to drive appetite in several animal populations, including humans [49,50]. While recent dietary fashions have increased the general availability of higher protein, non-home-prepared meals in Western countries, many of the young women in this study reported discretionary-style evenings meals, comprised of lower protein, high-carbohydrate foods, potentially leading to poor satiety, and increased drive to eat later in the evening after dinner. Furthermore, other behavioural and social factors are known to influence dietary choices, including but not limited to mood, social norms/culture, personal financial situation, and the quality and variety of the available foods/beverages [51,52]. Some of these have clearer one-way relationships with weight change, but others have potentially bidirectional of situationally relevant relationships with intake and weight. Future research should include consideration of these behavioural and social factors where possible and prioritise the collection of qualitative data in addition to quantitative data, potentially including discrete choice experiments to determine the influence of each factor in the context of the young person’s situation.

There are several limitations of this study, including its cross-sectional design and small convenience sample, which limited the capacity to conduct more robust statistical inference. The participants were also treatment-seeking young women aiming to lose weight and therefore may not be representative of the broader population of young adults with overweight and obesity. Qualitative data on participant demographics, including socio-economic status, mood, cultural backgrounds and beliefs, and pre-existing dietary requirements (e.g., vegetarian, coeliac), were not collected, all of which may have influenced dietary patterns, including diet quality [51,52,53,54]. Participants were not asked about their menstrual cycle, and while there is some evidence regarding the statistical and clinical significance of change in energy intake and bodyweight over the menstrual cycle, the effect of obesity on these factors is uncertain; future research should consider menstrual cycle phase to enable this to be accounted for as a potentially important covariate. Additionally, whilst participants were recruited across the year, timepoint of food diary completion in the year was not recorded, and therefore, accounting for seasonal differences to intake was not possible. The results were also reliant on self-reported data, which common to many nutrition studies, can be prone to under-reporting when it comes to nutrition and intake data [55]. The study also included limited qualitative data, which may have been useful for determining the reasoning behind the dietary choices (social, financial, due to insufficient cooking skills) and also the context surrounding the high number of meals not prepared at home (e.g., eaten in a restaurant or as takeaway and alone or in social situations). Even so, the study has highlighted the importance of considering additional lifestyle factors when developing weight loss interventions, especially for young adults, as they may pose effective and accessible solutions for this unique population.

## 5. Conclusions

This study has identified key dietary patterns present in young women with overweight and obesity, including later weighting of total energy intake, high intake of discretionary foods and beverages, and poor-diet quality. Future research is warranted to determine the impact of altering these dietary patterns on weight management in young adult populations. Furthermore, young adults tend to be neglected in weight management research with most interventions targeting older adults who have already developed obesity or metabolic diseases [9,40,56] and with such interventions also often not being suited to younger adults who face different challenges in what may be considered a comparatively volatile period of life changes. As such, there may be value in using the findings presented here, in addition to existing and future research that may arise as a result of this work, to support the development of tailored education and messaging for young adults at key settings within this transitional life stage, such as in the tertiary education setting, new workers, and when leaving the family home, when they are likely to have greater autonomy over their dietary choices and can independently put these lessons into practice. This is an important area for research and development in order that young adults and indeed young women may be better placed to prevent weight gain in this key transitional period of life.

## Figures and Tables

**Table 1:** Average intake by participants’ 3-day weighed food diary analysis.

**Timing of Eating or Drinking**	
First eating or drinking occasion (24-h time)	0912 (1 h 41 min)
Last eating or drinking occasion (24-h time)	2043 (1 h 17 min)
Eating or drinking window (hours, h)	11.5 (2.3)
Number of eating or drinking occasions (*n*)	4.8 (0.6)
Total energy (kJ/day)	9174 (2526)
**Percentage of total energy (% of total energy intake)**	
Fat	36 (6.8)
Saturated fat	17.8 (3.7)
Carbohydrates	39.8 (7.9)
Protein	19.4 (4.3)
Discretionary items	36.8 (16.8)
**Number of serves of each food group ^a^**	
Fruit	1 (0.97)
Vegetables	4 (2.38)
Dairy	1.56 (0.87)
Grains	6 (3)
Wholegrains (% of total grains)	29.0 (30.4)
Discretionary items	6 (4)
Takeaway meals/day	1 (range: 0–3)
Participants consuming > 1 takeaway meal/day *n* (%)	5 (18)
Sugar-sweetened beverages	1 (range: 0.2)
Non-nutritive-sweetened beverages	1 (range: 0–2)
Alcohol (standard drinks)	0.6 (1.1)
Iron (mg)	11.54 (5.78)
Calcium (mg)	804.8 (283.9)

^a^ serves calculated as per Australian Guide to Health Eating [25]; data are reported as mean (SD) or median (range).

## Data Availability

Data presented in this study are available on request from the corresponding authors.
